# Electrically Polarized Graphene-Blended Spacers for Organic Fouling Reduction in Forward Osmosis

**DOI:** 10.3390/membranes11010036

**Published:** 2021-01-04

**Authors:** Numan Yanar, Yejin Liang, Eunmok Yang, Hosik Park, Moon Son, Heechul Choi

**Affiliations:** 1 School of Earth Sciences and Environmental Engineering, Gwangju Institute of Science and Technology (GIST), 123-Cheomdangwagi-ro, Buk-gu, Gwangju 61005, Korea; numanyanar@gm.gist.ac.kr (N.Y.); liangyejin@gist.ac.kr (Y.L.); yang1990@gist.ac.kr (E.Y.); 2 Green Carbon Research Center, Chemical Process Division, Korea Research Institute of Chemical Technology (KRICT), Daejeon 34114, Korea; 3 School of Urban and Environmental Engineering, Ulsan National Institute of Science and Technology, 50, UNIST-gil, Eonyang-eup, Ulju-gun, Ulsan 44919, Korea

**Keywords:** organic fouling, electrically polarized spacer, 3D printed spacer, forward osmosis, graphene-blended spacers, nanomaterial-blended spacers

## Abstract

In membrane processes, a spacer is known to play a key role in the mitigation of membrane fouling. In this study, the effect of electric polarization on a graphene-blended polymer spacer (e.g., poly(lactic acid), PLA) for organic fouling on membrane surfaces was investigated. A pristine PLA spacer (P-S), a graphene-blended spacer (G-S), and an electrically polarized graphene-blended spacer (EG-S) were successfully fabricated by 3D printing. Organic fouling tests were conducted by the 5-h filtration of CaCl_2_ and a sodium alginate solution through commercially available membranes, which were placed together with the fabricated spacers. Membranes utilizing P-S, G-S, and EG-S were characterized in terms of the fouling amount on the membrane surface and fouling roughness. Electrostatic forces of EG-S provided 70% less and 90% smoother fouling on the membrane surface, leading to an only 14% less water flux reduction after 5 h of fouling. The importance of nanomaterial blending and polarization was successfully demonstrated herein.

## 1. Introduction

Membrane treatment methods have been playing a crucial role in providing potable water [1]. As a result of the depletion of fresh water sources due to global warming [2], these methods are gaining increasing importance. However, energy efficiency, which is directly related to membrane permselectivity, must be maintained well even after long-term use [3,4]. Fouling, concentration polarization, and mechanical damage of the membranes are the most critical factors that directly affect the membrane performance and energy efficiency [5,6,7,8]. Among these factors, membrane fouling in membrane systems is the most crucial issue that needs to be considered, especially because brackish water, wastewater and seawater comprise a number of foulants that can block the membrane surface and pores, thereby reducing the productivity of treated water [5,9].

Various types of membrane fouling include crystalline fouling, organic fouling, particle fouling, colloidal fouling, and biofouling [10,11]. Among these types, organic fouling serves as the major constraint due to the presence of relatively high concentrations of organics in water [12]. Organic fouling is a critical issue for all types of filtration membranes, including microfiltration (MF), ultrafiltration (UF), nanofiltration (NF), reverse osmosis (RO), and forward osmosis (FO). Organic fouling results from the aggregation of organic materials such as proteins, sugar, humic-acid-like substances and polysaccharides on the membrane surface [13,14]. Proteins creating organic fouling are present in a high ratio in wastewater, which aggregate on the membrane surface due to hydrogen bonding between the molecules [13]. This aggregation leads to severe fouling, which in turn decreases the performance, i.e., water production capacity.

FO membranes demonstrate immense potential and extensive applications to reduce the total energy consumption during desalination and waste water treatment. As a result of benefitting from the draw solution with a high osmotic concentration, FO systems can filter water with foulants without the use of any external pressure through high-pressure pumps [15]. As FO is responsible for handling water contaminants rather than desalting, organic fouling is highly critical for this system. Previously, Zhao et al. reported that organic fouling is more severe and irreversible in FO systems [16]. FO fouling results from effects of chemical and hydrodynamic interactions. A fouling layer is developed on the membrane surface by major factors, including calcium binding, permeation drag, and hydrodynamic shear force [17]. In this regard, approaches including the use of fouling-resistant novel membranes, change in the hydrodynamic conditions, use of membrane feed spacers, and pre/post-treatment are mainly employed to mitigate organic fouling. Among these approaches, feed spacers have been extensively investigated thus far as spacers for an FO membrane module are essential for maintaining a flow channel and providing hydrodynamic conditions [18]. An effective feed spacer also should work well for reducing foulant deposition and concentration polarization [19].

Various studies on feed spacers have focused on the spacer shape [19,20]. Previous studies employed computational fluid dynamics (CFD) to investigate the effect of spacer shapes, including nonwoven, woven, middle layer, and fully woven spacers [21]; 30°, 45°, 62°, and 90° spacer filaments [22]; hairy spacers [23]; saw-tooth spacers [24]; zigzag spacers [25]; multi-layer spacers [26]; and sinusoidal spacers [27], on the membrane performance. In addition, with the boost of 3D printing, the design of spacers was affected [28,29], i.e., column-type spacers [30], triply periodic minimal surfaces (TPMS) spacers [31], symmetric perforated spacers (1-Hole, 2-Hole, and 3-Hole) [32], and honeycomb spacers [33], were fabricated by 3D printing. In addition, 3D printing provides material selection for spacer fabrication. Previously, our group investigated the performance of 3D printed spacers comprising acrylonitrile-butadiene-styrene, (poly(lactic acid), PLA), and polypropylene. Spacers composed of different materials exhibit different performances [34]. Benefitting from recent developments in 3D printing, our group also recently introduced a graphene-blended membrane spacer and investigated the effect of electric polarization on the graphene-blended spacer [35]. Through that work, we reported the performance of the electrically polarized graphene-blended spacer as a draw spacer for flux enhancement, and as a feed spacer for membrane scaling. Apart from scaling, organic fouling is also a critical issue that has to be addressed due to its sticky nature. By considering the importance of mitigating organic fouling of FO membranes, the electrically polarized graphene spacer was further investigated in this study. To the best of our knowledge, this is the first study of an electrically polarized graphene-blended polymer spacer for the mitigation of organic fouling.

## 2. Materials and Methods

**Figure 1:**
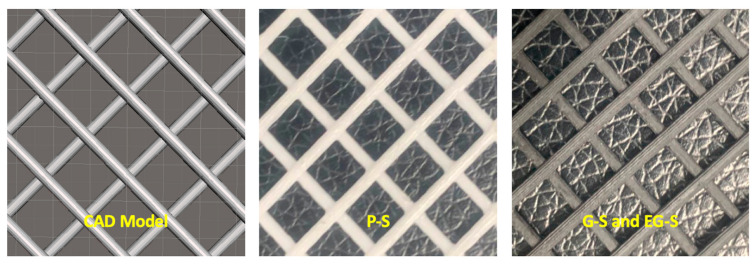
CAD model; 3D printed poly(lactic acid) (P-S); graphene-blended poly(lactic acid) (G-S); and electrically polarized graphene-blended poly(lactic acid) (EG-S).

Three fabricated spacers prepared by 3D printing after computer-aided design (CAD) modelling were used for experiments. One PLA and two graphene-blended PLA spacers were fabricated by using the same structural parameters (i.e., spacer filament thickness of 1.27 mm, with a vertical and horizontal spacer hole distance of 7 mm) (Figure 1). All spacers were fabricated by using a fused deposition modelling (FDM)-type 3D printer (OpenCreators-Almond, Gyeonggi, Korea). PLA spacer was fabricated by using a PLA filament (PLABS, Gyeonggi, Korea) which has 1.24 gm/cm^3^ specific gravity, 26.4 MPa tensile strength, 2.3 GPa tensile modulus and elongation break at 4%, and graphene-blended PLA spacers were fabricated by using conductive graphene blended PLA filaments which has 1.11 gm/cm^3^ specific gravity, 53 MPa tensile strength, 2.9 GPa tensile modulus and elongation break at 5.1% (Graphene Laboratories, Ronkonkoma, NY, USA) [36]. Later, one of the graphene-blended PLA spacers was electrically polarized under an electric field of 1.5 kV/cm for 2 h. Further details regarding the fabrication of the spacers by using FDM type 3D printer as well as electric polarization were reported in another article by our group [35]. In this article, PLA, graphene-blended poly(lactic acid), and electrically polarized graphene-blended PLA spacers were denoted as P-S, G-S, and EG, respectively. 

**Figure 2:**
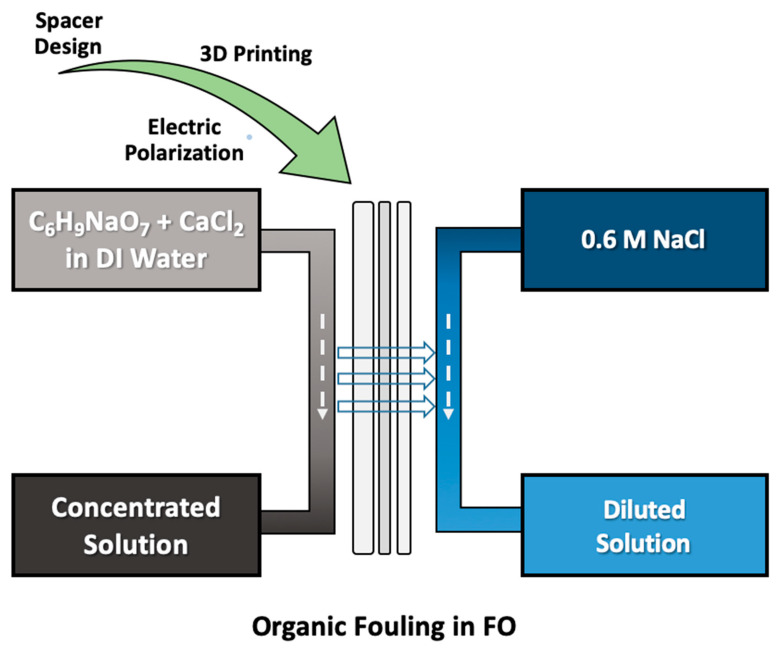
Organic fouling test of 3D printed spacers through a forward osmosis system.

Flux measurements were conducted for pure water flux/reverse solute flux measurements and fouling-based flux reduction. For the performance investigation of the spacers, commercial membranes (Porifera, San Leandro, CA, USA) were used, which were extracted from a commercial membrane module. An engineered osmosis system (Figure S1) with an effective filtration area of 19.35 cm^2^ was utilized in the continuous filtration mode at a cross flow rate of 200 cm^3^/s for the draw and feed sides (Figure 2). For the pure water flux and reverse solute flux measurements, a 0.6 M NaCl (Sigma-Aldrich, Burlington, MA, USA) solution was prepared as the draw solution, and deionized (DI) water (Synergy, Millipore, Billerica, MA, USA) with a resistivity of 18.2 mΩ-cm at 25 °C was used as the feed solution. Filtration was conducted for 1 h for each sample, with 5 times repetition. For the fouling experiment, an alginate-based feed solution was prepared, while 0.6 M NaCl was used as the draw solution. As the fouling solution, 200 ppm of C_6_H_9_NaO_7_ (sodium alginate, Sigma-Aldrich, Burlington, MA, USA) and 1 mM of CaCl_2_ (Sigma-Aldrich, Burlington, MA, USA) were dissolved in DI water [37]. Here, CaCl_2_ was used as the Ca^2+^ source to bind alginate molecules. Filtration was conducted for 5 h after flux stabilization was performed for 1 h. Data obtained by the weight change in the feed solution in pure flux and fouling experiments, as well as feed conductivity for only pure flux experiments, were recorded every minute by a connected computer. From these recorded values, the water flux, J_w_, and the reverse solute flux, J_s_, were calculated by using the following formulas [38,39]:

(1) $$J_{w}=\frac{V}{A_{m}\Delta t}$$

(2) $$J_{s}=\frac{V_{t}C_{t}-V_{0}C_{0}}{A_{m}t}$$

In the first formula, J_w_ is the water flux, V is the volume of permeated water (L), A_m_ is the effective membrane area (m^2^), and Δt is the permeation time (min). The reverse solute flux was calculated from the electrical feed conductivity per minute [39]. In the second formula, C_t_ is the concentration (g/L) at time t, V_t_ is the volume (L) of the feed solution at time t, C_0_ is the initial concentration (g/L), and V_0_ the initial volume (L) of the feed solution. The resulting values were converted into Lm^−2^h^−1^ (LMH) for water flux and gm^−2^h^−1^ (gMH) for reverse solute flux.

Surface roughness of fouled membranes, foulant volume on membranes, and visual topographic images of fouled membranes were obtained by a Surface Nano-Profiler (Nanomap-D/Alpha-steps, HTSK, Gyeonggi, Korea) measurements. 3D measurements were performed with an x–y scanning distance of 500 µm and a scanning speed of 50 µm/s at 10 steps for each sample. These measurements were repeated 10 times for each sample. Notably, characterization was performed for surface areas that were not in contact with the spacers.

The amounts of foulant adsorbed on the G-S and EG-S spacers were investigated by dipping the spacers in 200 mL of an alginate solution for 12 h (same solution as mentioned above) at room temperature. Spacer weights were measured before and after the dipping process (after drying in an oven at 40 °C for 2 h). Later, spacers were cleaned in an ultrasonicator (B8510-MT, Branson, Brookfield, CT, USA) by dipping in DI water. After cleaning in an oven at 40 °C for 2 h, the spacer weight was measured again. Finally, the amounts of foulant adsorbed on G-S and EG-S spacers (before and after cleaning) were obtained. Notably, the resistance of spacers against heating and sonication was repetitively tested by using non-fouled spacers. G-S and EG-S spacers did not exhibit any deformation, while the P-S spacer exhibited weight loss and structural deformation. Therefore, the P-S spacer is not included in this investigation.

## 3. Results and Discussion

**Figure 3:**
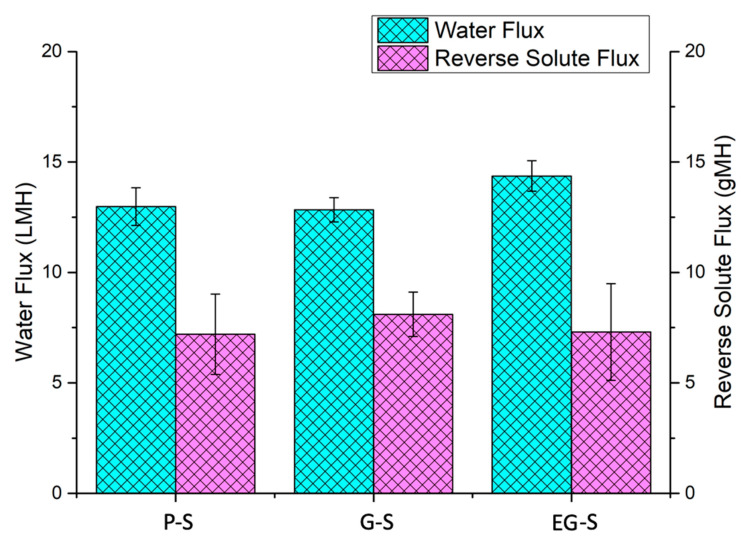
Water fluxes and reverse solute fluxes for P-S, G-S, and EG-S spacers.

P-S and two G-S spacers were successfully printed, and one of the G-S spacers was polarized to obtain an electrically polarized EG-S spacer. All three samples were examined for pure water flux and reverse solute flux measurements. Flux performances of all three spacers were relatively similar. More specifically, P-S, G-S, and EG-S exhibited water flux of 13 ± 0.85 LMH, 12.8 ± 0.55, and 14.4 ± 0.69 LMH, respectively (Figure 3). In addition, similar values for reverse solute fluxes were observed: 7.2 ± 1.8 gMH for P-S, 8.1 ± 1 gMH for G-S, and 7.3 ± 2.2 gMH for EG-S (Figure 3). Marginal changes in the flux values were attributed to the same structure of spacers. 

**Figure 4:**
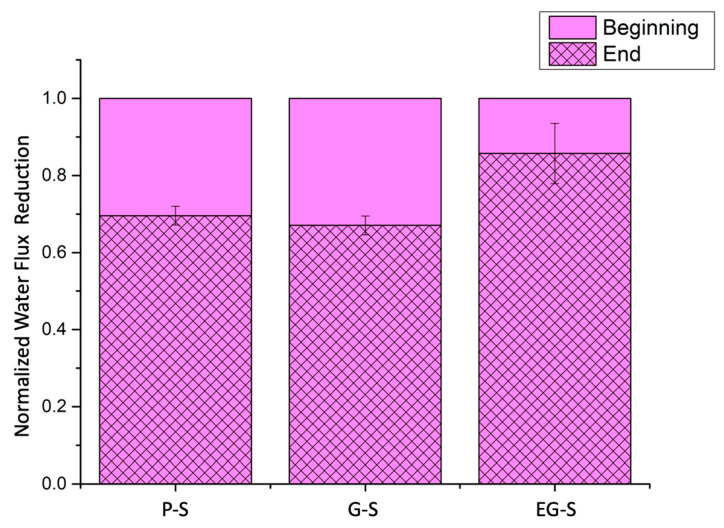
Normalized water flux reduction for the P-S, G-S, and EG-S membranes.

Additional tests were performed to investigate the organic fouling performance of P-S, G-S, and EG-S spacers. After 5 h of alginate-based fouling of membrane spacers, the fouled membranes with P-S or G-S exhibited a water flux reduction of 31% or 33%, respectively (Figure 4). This ratio was found to be 14% of the water flux for the fouled membranes with the EG-S spacer. Even though graphene blending did not considerably affect the performance of the PLA spacer for water flux reduction, the electric polarization of the graphene-blended spacer was highly effective in preventing the water flux reduction of the EG-S membrane. 

**Figure 5:**
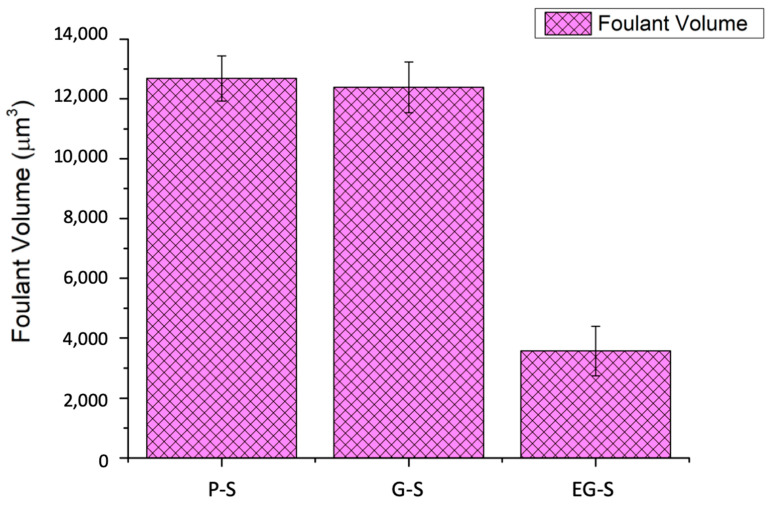
Average foulant volume for the fouled membranes with the P-S, G-S, and EG-S spacers.

**Figure 6:**
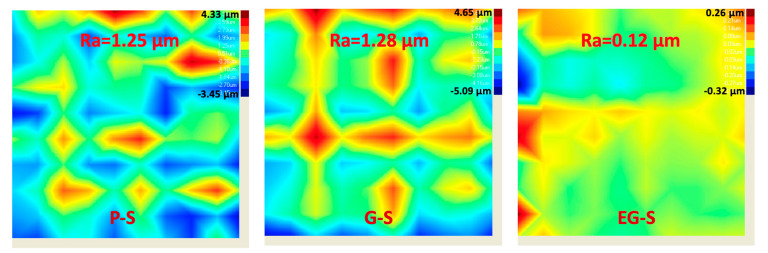
Surface profiles of the P-S, G-S, and EG-S fouled membranes (Roughness values also are provided on each figure). (Blue and red colors represent low fouling and high fouling thicknesses, respectively).

To understand the fouling phenomena, fouling characterization of the P-S, G-S, and EG-S fouled membranes was further performed using the Surface Nano-profiler via the analysis of the foulant volume and roughness, and representative images were obtained. The Surface Nano-profiler permitted the characterization of fouling in a larger area compared to other topographic fouling characterization methods. Results obtained from the surface nano-profiler also supported the data obtained from water flux reduction. On a 500 × 500 µm^2^ area, P-S, G-S, and EG-S membranes exhibited 12,685 ± 759 µm^3^, 12,391 ± 848 µm^3^, and 3570 ± 824 µm^3^ foulant, respectively, from an average of 10 measurements for each membrane (Figure 5). The membrane with the EG-S spacer exhibited 70% less foulant on its surface. The presence of Ca^2+^ in the fouling solution induces bridges between the negatively charged alginate molecules [40,41], resulting in positively charged Ca-alginate bridges [42,43]. That is, positive Ca^2+^ binds to negatively charged carboxylic groups of alginate [44], which is defined as the egg-box model in literature to explain the interaction between polysaccharides and divalent cations [45]. This generates aggregation as well as a compact fouling layer on the membrane surface [46]. Hence, roughness of fouled membranes is observed. Roughness (Ra) values of 1.25 µm, 1.28 µm, and 0.12 µm were obtained for the P-S, G-S, and EG-S fouled membranes, respectively. In addition, the smooth fouled surface of EG-S as well as rough fouled surfaces of P-S and G-S were observed in the representative characterization images from the surface nano-profiler (Figure 6). Here, the surfaces can be explained by the manipulation of a positively charged Ca-alginate bridge and physically bonded Ca^2+^ by the EG-S electrostatic forces. In a previous study reported by our group, the polarized spacer was found to exhibit dominating negative charges [35]. Therefore, positively charged Ca-alginate bridges are readily deposited on the negatively charged spacers. Furthermore, EG-S reduces the physical binding effect of Ca^2+^ between alginate molecules, and the aggregation of alginate molecules is less on the membrane surface, which can be understood from the lower roughness value of the EG-S membrane. Hence, fouling on the EG-S membrane is less than that on the P-S and G-S membranes. 

**Figure 7:**
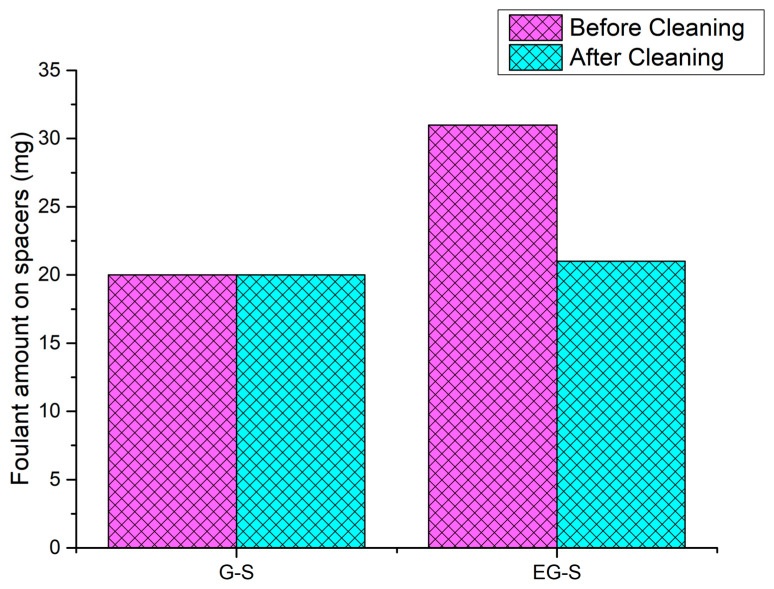
Efficacy of electric polarization on the adsorption of foulants on the G-S and EG-S spacers.

By considering the spacer fouling issue, the effect of electric polarization was further investigated by measuring the total adsorbed foulant amount on the spacers G-S and EG-S. After dipping the G-S and EG-S spacers for 12 h, 20 mg and 31 mg foulants were adsorbed, respectively. After the cleaning process, both spacers exhibited the same amount of foulant on the surface (20 mg) (Figure 7). In here, electric polarization permitted the deposition of the foulant on EG-S with weaker adhesion, leading to a lower foulant deposition on the EG-S membrane. 

## 4. Conclusions

In this article, the performance of electrically polarized graphene-blended poly(lactic acid) spacer (EG-S) was analyzed for alginate fouling. Compared to P-S and G-S, EG-S exhibited 70% less fouling on the membrane surface with a smoother fouling layer. Therefore, only 14% water flux reduction ratio was observed for the membrane of EG-S, while it was 31% or 33% for the membranes of P-S or G-S, respectively. This EG-S performance was attributed to the electrostatic manipulation of Ca-alginate bridges and Ca^2+^ ions which are binding alginate molecules. Regarding to this, foulant adsorption test G-S and EG-S also showed that EG-S can collect 55% more foulant on itself than G-S, and it was also investigated that this additional amount of foulant collected by the effect of electric polarization is cleanable. However, this point should be extensively studied further in another research. For future studies, high-pressure membrane systems such as NF or RO also should be considered by utilizing various organic foulants such as humic acid or bovine serum albumin.

## Data Availability

Not applicable.

## Supplementary Materials

The following are available online at https://www.mdpi.com/2077-0375/11/1/36/s1, Figure S1: Engineered osmosis system.
