# Supplementary material for: Electrically Polarized Graphene-Blended Spacers for Organic Fouling Reduction in Forward Osmosis
Source: Membranes (Basel). 2021 Jan 4;11(1):36. doi: 10.3390/membranes11010036 (PMC7823477; doi:10.3390/membranes11010036)
Supplement: Supplementary file 1 [file membranes-11-00036-s001.pdf]

Supporting Information

Electrically Polarized Graphene-blended Spacers for Organic Fouling Reduction in Forward Osmosis

Numan Yanar [1], Yejin Liang [1], Eunmok Yang [1], Hosik Park [2,*], Moon Son [3,*] and Heechul Choi [1,*]

1 School of Earth Sciences and Environmental Engineering, Gwangju Institute of Science and Technology (GIST), 123-Cheomdangwagi-ro, Buk-gu, Gwangju 61005, Korea; numanyanar@gm.gist.ac.kr (N.Y.); liangyejin@gist.ac.kr (Y.L); yang1990@gist.ac.kr (E.Y.)

2 Green Carbon Research Center, Chemical Process Division, Korea Research Institute of Chemical Technology (KRICT), Daejeon 34114, Korea

3 School of Urban and Environmental Engineering, Ulsan National Institute of Science and Technology, 50, UNIST-gil, Eonyang-eup, Ulju-gun, Ulsan 44919, Korea

* Correspondence: Correspondence: hcchoi@gist.ac.kr (H.C.); moonson619@unist.ac.kr (M.S.); hspark@krict.re.kr (H.P.); Tel.: +82-62-715-2441; Fax: +82-62-715-2423 (H.C.)

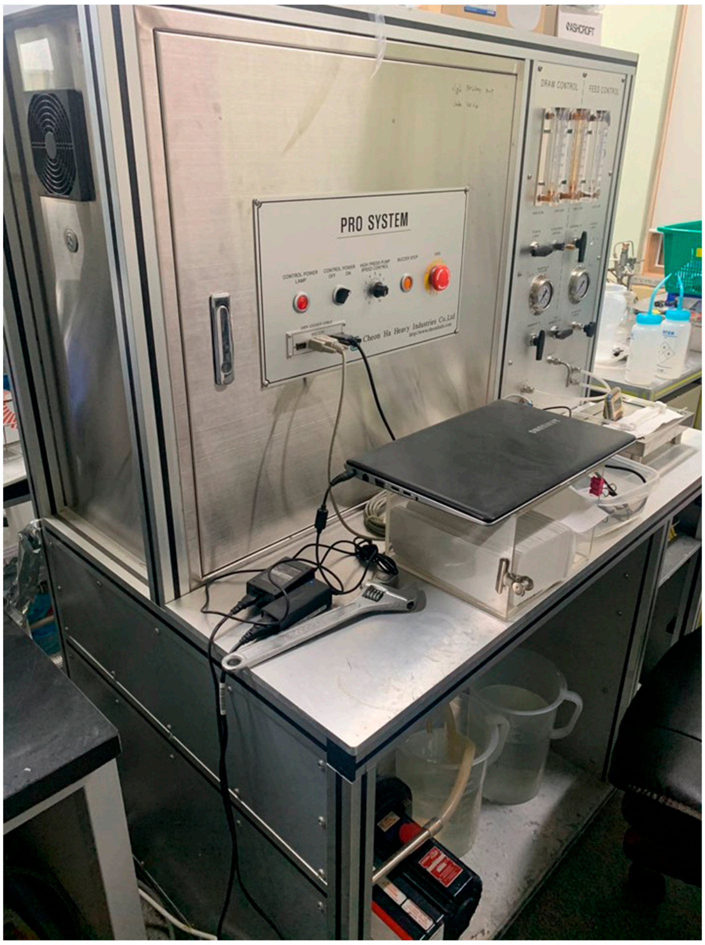

Figure S1. Engineered osmosis system.
